# Therapeutic Algorithm for Extramammary Paget Disease: Experience in a Dermatology Referral Center in Western Mexico

**DOI:** 10.7759/cureus.101408

**Published:** 2026-01-12

**Authors:** Michelle Alcocer Salas, Diana L Vazquez-Cantu, Mercedes Hernández Torres, Víctor Manuel Tarango-Martinez

**Affiliations:** 1 Dermatology, Instituto Dermatológico de Jalisco "Dr. José Barba Rubio", Guadalajara, MEX; 2 Dermatopathology, Instituto Dermatológico de Jalisco “Dr. José Barba Rubio”, Guadalajara, MEX

**Keywords:** epem, extramammary paget disease, immunohistochemistry (ihc), ­skin cancer, treatment algorithm

## Abstract

Introduction

Extramammary Paget disease (EMPD) is a rare intraepithelial adenocarcinoma that predominantly affects areas rich in apocrine glands. Its chronic and nonspecific clinical presentation frequently leads to misdiagnosis and delayed treatment.

Objective

The primary objective is to describe the clinical, epidemiological, histopathological, and therapeutic characteristics of patients with EMPD treated at a dermatology referral center. The secondary outcome is to propose a diagnostic and therapeutic algorithm based on institutional experience.

Methods

A single-center, retrospective, observational case series was conducted at the Instituto Dermatológico de Jalisco. All patients with histopathologically confirmed EMPD diagnosed between 2011 and 2025 were included. Demographic, clinical, histopathological, immunohistochemical, and therapeutic data were analyzed. Immunohistochemistry and staging studies were performed selectively based on clinical suspicion of secondary disease.

Result

Ten cases were identified (N = 10). There was a marked female predominance (90%), with a median age of 69 years (range: 57-86). Lesions were primarily located on the vulva (70%), followed by the perianal region (20%) and scrotum (10%). All patients presented with chronic, well-demarcated erythematous plaques. The median diagnostic delay was 20 months (range: 2-60). Immunohistochemistry was performed in six patients (60%), all demonstrating a CK7-positive/CK20-negative profile, confirming primary EMPD in all cases. No associated internal malignancies were identified. All patients underwent wide-margin surgical excision.

Conclusions

EMPD requires high clinical suspicion and early biopsy of persistent genital or perianal lesions. All cases in this series corresponded to primary EMPD with no associated malignancy. Surgical excision remains the standard of care. The proposed algorithm provides a practical, experience-based framework to guide diagnostic evaluation and management in referral centers.

## Introduction

Extramammary Paget disease (EMPD) is a rare intraepithelial adenocarcinoma that predominantly arises in areas rich in apocrine glands, including the vulva, perianal region, scrotum, and less commonly the axillae and umbilicus. It is an uncommon malignancy, with an estimated incidence of approximately 0.12 cases per 100 million individuals annually, underscoring its extreme rarity and the limited availability of large prospective studies [1].

Clinically, EMPD typically presents as chronic, persistent, well-demarcated erythematous plaques, frequently accompanied by scaling, ulceration, crusting, bleeding, or pigmentary changes. Symptoms such as pruritus, burning sensation, and pain are common, although up to 10% of patients may remain asymptomatic [2]. Due to its nonspecific presentation, EMPD is frequently misdiagnosed as more common inflammatory dermatoses, including eczema, psoriasis, candidiasis, or erosive lichen planus, leading to substantial diagnostic delay and prolonged morbidity [2].

Histopathological examination remains the cornerstone of diagnosis, demonstrating characteristic large pale Paget cells within the epidermis. Immunohistochemistry (IHC) plays an important adjunctive role, particularly in distinguishing primary cutaneous EMPD from secondary disease associated with underlying internal malignancies. A cytokeratin 7 (CK7)-positive and cytokeratin 20 (CK20)-negative profile supports a primary cutaneous origin, whereas CK20 positivity may suggest secondary involvement from gastrointestinal or urothelial primaries [3,4].

Management of EMPD remains challenging due to its high recurrence rates and potential association with internal malignancies. Surgical excision with wide margins remains the standard of care, although radiotherapy, topical therapies, and systemic treatments have been reported in selected cases, particularly when surgery is not feasible or margins are positive [5].

Given the rarity of EMPD, most available evidence derives from small institutional case series. Studies conducted in dermatology referral centers are particularly valuable, as they provide real-world insight into diagnostic challenges, treatment patterns, and outcomes in specialized clinical settings. The present study aims to describe the clinical, histopathological, and therapeutic characteristics of patients with EMPD treated at a tertiary dermatology referral center in western Mexico. Additionally, we propose a pragmatic diagnostic and therapeutic algorithm based on institutional experience to assist clinicians in the evaluation and management of this uncommon malignancy.

## Materials and methods

Study design and setting

This was a single-center, retrospective, observational case series conducted at the Instituto Dermatológico de Jalisco, a tertiary dermatology referral center in western Mexico.

Study population

All patients diagnosed with EMPD between January 2011 and May 2025 were identified through the institutional histopathology database and clinical records.

Inclusion Criteria

All cases with histopathological confirmation of EMPD were included in the study.

Exclusion Criteria

No exclusion criteria were applied.

Data collection

Clinical and pathological information was obtained from electronic and physical medical records. Variables collected were grouped as follows: demographic variables: age and sex; clinical variables: lesion location, morphology, symptoms, duration of disease, and initial clinical diagnosis; pathological variables: histopathological findings; IHC variables: CK7 and CK20 expression; evaluation of associated malignancy: staging investigations; and therapeutic variables: treatment modality and surgical margins.

Histopathology and IHC

All diagnoses were confirmed by histopathological examination, identifying characteristic intraepidermal Paget cells. IHC studies were performed selectively in cases with clinical or histopathological suspicion of secondary EMPD and were not applied routinely to all patients. The primary markers used were CK7 and CK20 to differentiate primary from secondary disease. Additional markers (e.g., GCDFP-15 and HER2) were not required due to consistent immunophenotypic profiles.

Staging and evaluation of associated malignancy

Staging investigations were requested only when clinically indicated and included gynecologic examination and Papanicolaou smear, colonoscopy, cystoscopy, abdominopelvic computed tomography (CT), and tumor markers (carcinoembryonic antigen (CEA), carbohydrate antigen (CA) 19-9, and CA 15-3). These assessments were performed to rule out underlying or associated malignancies in cases with suspected secondary EMPD.

Treatment

Treatment decisions were individualized based on lesion size and anatomical location, histopathological findings, surgical margin status, patient comorbidities and functional status, and institutional surgical protocol. All patients underwent wide-margin surgical excision, with margins ranging from 1 to 2 cm, in accordance with institutional practice and published recommendations.

Ethical considerations

This study was conducted in accordance with the Declaration of Helsinki and its later amendments. Due to its retrospective nature, formal approval from the institutional ethics committee was waived. All patient data were anonymized to ensure confidentiality.

Statistical analysis

Descriptive statistics were used. Categorical variables are presented as frequencies and percentages, and continuous variables as medians with ranges.

## Results

A total of 10 patients with EMPD were included in the study (N = 10). There was a marked female predominance, with nine women (90%) and one man (10%), yielding a female-to-male ratio of 9:1. The median age at diagnosis was 69 years (range: 57-86 years).

Clinical characteristics

Lesions were most frequently localized to the vulva in seven patients (70%), followed by the perianal region in two patients (20%) and the scrotum in one patient (10%). All patients presented with chronic, well-demarcated erythematous plaques. Additional clinical features included ulceration, maceration, scaling, vesiculation, bleeding, and pigmentary changes. Pruritus and burning sensations were the most commonly reported symptoms, although four patients (40%) were asymptomatic at presentation.

The duration of symptoms prior to diagnosis ranged from two to 60 months. The median diagnostic delay was 20 months, reflecting a substantial delay in clinical recognition in several cases.

Initial clinical suspicion

Initial clinical diagnoses before histopathological confirmation included inflammatory dermatoses in six patients (60%), such as contact dermatitis, psoriasis, erosive lichen planus, and lichen sclerosus. In the remaining four patients (40%), neoplastic conditions were suspected, including squamous cell carcinoma, basal cell carcinoma, melanoma, and EMPD. One patient (10%) had a documented previous history of vulvar carcinoma.

Histopathology and IHC

Histopathological examination confirmed EMPD in all cases (100%), demonstrating characteristic intraepidermal Paget cells. IHC analysis was performed in six patients (60%) based on clinical or histopathological suspicion of possible secondary disease. All tested cases demonstrated a CK7-positive/CK20-negative immunophenotype, supporting a primary cutaneous origin. Therefore, all cases in this cohort were classified as primary EMPD. Additional markers, including GCDFP-15 and HER2, were not required, as the CK7/CK20 profile was sufficient for diagnostic classification.

Evaluation for associated malignancy

Staging investigations were carried out in the same six patients who underwent IHC evaluation. These included gynecologic examination with Papanicolaou smear, colonoscopy, cystoscopy, abdominopelvic CT, and tumor marker analysis. No underlying or associated internal malignancies were identified in any patient.

Treatment

All patients (100%) underwent wide-margin surgical excision as primary treatment, with surgical margins ranging from 1 to 2 cm. Patients with positive margins were referred for re-excision or considered for adjuvant radiotherapy according to institutional protocol.

Summary of cases

The detailed clinical, histopathological, and therapeutic characteristics of all patients are summarized in Table 1. The heterogeneity in clinical presentation and frequent initial misdiagnosis highlight the diagnostic challenges associated with EMPD and emphasize the importance of early biopsy in persistent genital or perianal lesions.

**Table 1:** Clinical, Histopathological, and Treatment Characteristics of Patients With Extramammary Paget Disease (EMPD) SCC: squamous cell carcinoma

Case	Age (years)	Sex	Topography	Morphology	Evolution (months)	Clinical symptoms	Initial diagnostic suspicion	Histopathology	Associated neoplasia	Immunohistochemistry	Treatment
1	86	F	Vulva	Erythematous ulcerated plaque	48	Pruritus	SCC vs. erosive lichen planus vs. vulvitis	Intraepidermal Paget cells	No	CK7+, CK20-	Surgical
2	63	F	Suprapubic	Erythematous plaque with scaling and blue pigment	18	None	Basal cell carcinoma	Intraepidermal Paget cells	No	Not performed	Surgical
3	66	F	Vulva and perineum	Erythematous plaque with excoriations	12	Pruritus	EMPD	Intraepidermal Paget cells	No	Not performed	Surgical
4	73	F	Vulva	Whitish plaques	6	None	EMPD	Intraepidermal Paget cells	History of vulvar carcinoma	Not performed	Surgical
5	60	F	Vulva	Ulcerated and bleeding neoformations	12	Pain	Lichen sclerosus	Intraepidermal Paget cells	No	CK7+, CK20-	Surgical
6	81	F	Vulva	Macerated plaques	60	None	Lichen sclerosus	Intraepidermal Paget cells	No	CK7+, CK20-	Surgical
7	57	F	Perineum	Ulcerated erythematous neoformation	12	None	Melanoma vs. basal cell carcinoma	Intraepidermal Paget cells	No	CK7+, MELAN-A-	Surgical
8	66	F	Vulva and perineum	Hyperkeratotic plaque with hypo-/hyperpigmentation	4	None	EMPD vs. Bowen disease	Intraepidermal Paget cells	No	CK7+, CK20-	Surgical
9	72	F	Vulva	Erythematous plaque with vesicles and ulceration	24	None	Pemphigus vulgaris vs. erosive lichen planus vs. EMPD	Intraepidermal Paget cells	No	CK7+, CK20-	Surgical
10	73	M	Scrotum	Erythematous and scaly plaque	1	None	Psoriasis vs. contact dermatitis	Intraepidermal Paget cells	No	Not performed	Surgical

## Discussion

EMPD remains a rare cutaneous malignancy associated with significant diagnostic and therapeutic challenges. In this single-center case series from a dermatology referral center, we describe the clinical and pathological characteristics of 10 patients and propose an experience-based diagnostic and therapeutic algorithm. Our cohort demonstrated a marked female predominance (90%) and a median age at diagnosis of 69 years, findings consistent with previously published series reporting a higher prevalence among elderly women [6,7].

The vulva was the most commonly affected site (70%), followed by the perianal region (20%) and scrotum (10%). This anatomical distribution closely mirrors reports in the literature, where vulvar involvement accounts for approximately 65%-70% of cases [6,8], supporting the external validity of our findings despite the inherent limitations associated with small sample sizes in rare diseases.

From a clinical perspective, EMPD continues to pose diagnostic difficulty due to its nonspecific presentation. In our series, 60% of patients were initially misdiagnosed with inflammatory dermatoses, including lichen sclerosus, erosive lichen planus, and contact dermatitis. This misdiagnosis rate aligns with previously reported figures ranging from 40% to 70% [7,9]. The median diagnostic delay of 20 months reflects prolonged symptom duration prior to biopsy. Although this delay is shorter than the up to 43 months described in other studies [9,10], it remains clinically significant and highlights the need for heightened clinical suspicion and early biopsy of persistent anogenital plaques.

Histopathological examination confirmed the diagnosis in all cases. IHC was selectively employed in patients with suspicion of secondary disease. All tested cases demonstrated a CK7-positive and CK20-negative immunophenotype, confirming a primary cutaneous origin. Consequently, all cases in this cohort were classified as primary EMPD. This finding is clinically relevant, as secondary EMPD is associated with underlying internal malignancies and requires a distinct diagnostic approach [10,11].

Staging investigations performed in selected patients revealed no associated internal malignancies. This is an important observation, as it supports evidence suggesting lower rates of associated neoplasia in vulvar-predominant cohorts compared with perianal or male genital involvement [6,8,12]. The absence of secondary malignancies in our cohort reinforces the importance of individualized staging based on clinical and histopathological features rather than routine extensive screening in all patients.

Surgical excision with wide margins remains the standard of care and was performed in all patients in this series. However, recurrence rates remain high even after apparently complete excision, as consistently reported in the literature [10-12]. For this reason, adjuvant therapies, including radiotherapy and topical treatments, may be considered in selected cases, particularly in patients with positive margins or contraindications to surgery.

**Figure 1:**
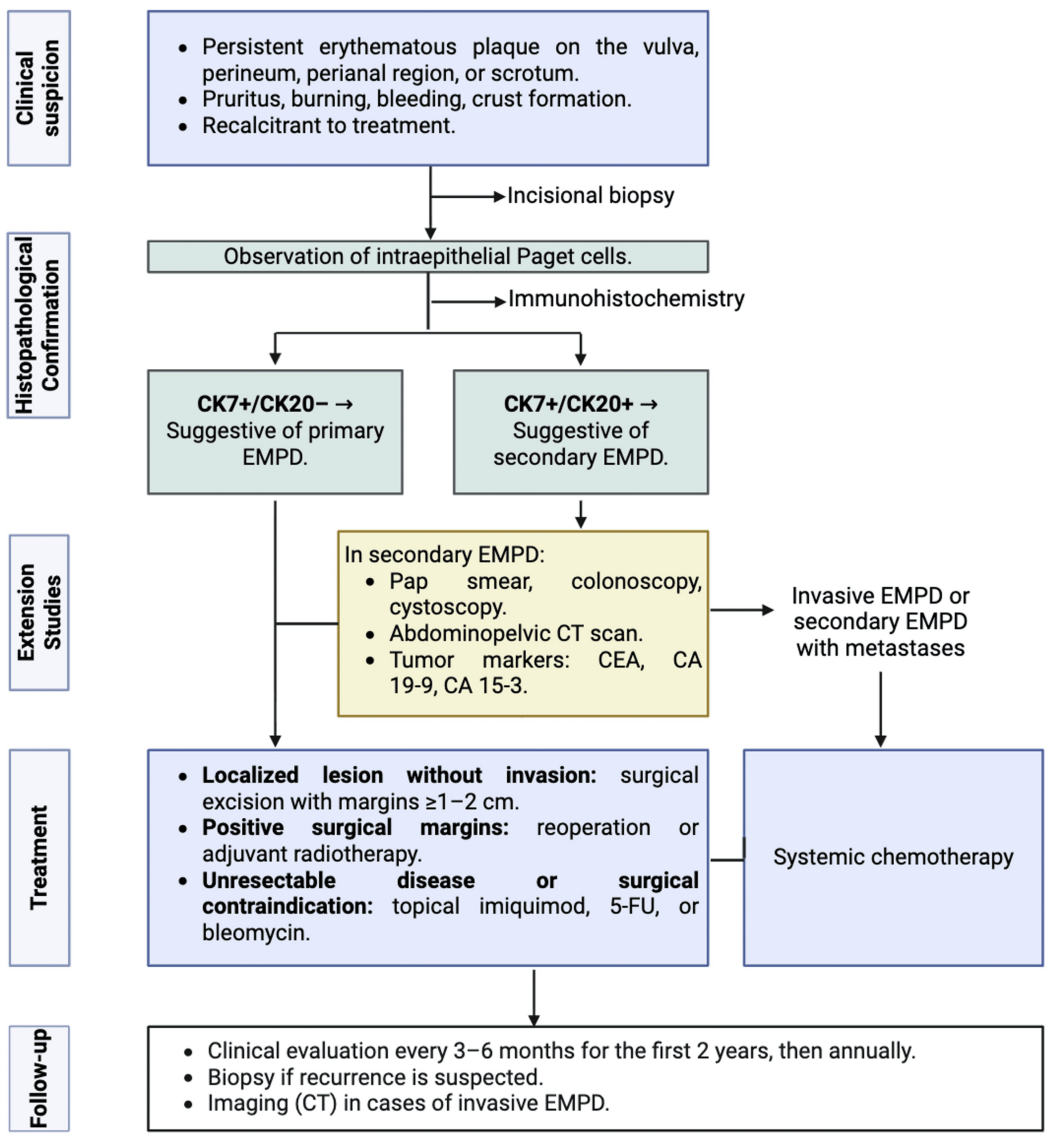
Diagnostic and Therapeutic Algorithm for Extramammary Paget Disease (EMPD) CT: computed tomography; CEA: carcinoembryonic antigen; CA: carbohydrate antigen; 5-FU: 5-fluorouracil

The principal contribution of this study is the translation of institutional experience into a pragmatic diagnostic and therapeutic algorithm (Figure 1). By integrating early clinical suspicion, prompt histopathological confirmation, selective use of IHC, and individualized staging, this framework provides a structured approach to management in referral centers. Importantly, this algorithm should be interpreted as hypothesis-generating and experience-based rather than prescriptive or evidence of therapeutic superiority.

This study has several limitations. The small sample size, retrospective design, and single-center setting limit generalizability. Additionally, long-term outcomes such as recurrence rates, disease-free survival, and standardized follow-up were not evaluated. Furthermore, the proposed algorithm has not been formally validated or compared with existing management strategies, which should be considered when interpreting these findings.

Despite these limitations, this series provides valuable real-world data from a specialized referral center and contributes to the growing body of literature on this rare malignancy. Future multicenter prospective studies with standardized outcome measures are needed to validate diagnostic pathways, optimize treatment strategies, and refine follow-up protocols.

## Conclusions

EMPD is an uncommon malignancy with a chronic and nonspecific clinical presentation that frequently leads to diagnostic delay and initial mismanagement. Early clinical suspicion and prompt biopsy of persistent genital or perianal lesions are essential to achieve a timely and accurate diagnosis. In this single-center case series, all patients were classified as having primary EMPD, and no associated internal malignancies were identified, underscoring the importance of selective rather than routine extensive staging based on clinical and histopathological features. Surgical excision with wide margins remains the standard of care and was successfully implemented in all cases.

The diagnostic and therapeutic algorithm proposed in this study provides a structured, experience-based framework to guide clinical decision-making in referral centers. However, this approach should be interpreted as hypothesis-generating rather than prescriptive. Future multicenter prospective studies with standardized outcome measures are needed to validate this strategy and to optimize diagnostic pathways, treatment selection, and follow-up protocols for patients with EMPD.
